# Application of multiplex amplicon deep-sequencing (MAD-seq) to screen for putative drug resistance markers in the *Necator americanus* isotype-1 β-tubulin gene

**DOI:** 10.1038/s41598-022-15718-1

**Published:** 2022-07-06

**Authors:** Santosh George, Peter Suwondo, Jewelna Akorli, Joseph Otchere, Lisa M. Harrison, Kaya Bilguvar, James R. Knight, Debbie Humphries, Michael D. Wilson, Adalgisa Caccone, Michael Cappello

**Affiliations:** 1 Department of Pediatrics, Yale School of Medicine, New Haven, CT 06520 USA; 2 Department of Parasitology, Noguchi Memorial Institute for Medical Research, University of Ghana, Legon, Ghana; 3 Yale Center for Genome Analysis, Yale School of Medicine, New Haven, CT USA; 4 Department of Chronic Disease Epidemiology, Yale School of Public Health, New Haven, CT USA; 5 Department of Ecology and Evolutionary Biology, Yale University, New Haven, CT USA

**Keywords:** High-throughput screening, Microbiology techniques, Sequencing

## Abstract

Global control of hookworm infections relies on periodic Mass Drug Administration of benzimidazole drugs to high-risk groups, regardless of infection status. Mutations in the isotype-1 β-tubulin gene have been identified in veterinary nematodes, resulting in structural changes and reduced drug-binding. In Ghana, previous studies have demonstrated significant variability in albendazole effectiveness among people infected with the hookworm *Necator americanus*, although the mechanisms underlying deworming response have not been defined. Using hookworm egg samples from a cross-sectional study in Ghana, we developed a multiplex amplicon deep sequencing (MAD-seq) method to screen genomic regions encapsulating putative drug-resistance markers in *N. americanus* isotype-1 β-tubulin gene. Three single nucleotide polymorphisms (SNPs) corresponding to resistance-associated mutations (F167Y, E198A, F200Y) within the coding region of the isotype-1 β-tubulin gene were characterized using MAD-seq in 30 matched pre- and post-treatment samples from individuals with persistent infection following therapy. Post-sequence analysis showed that the highest mean alternative nucleotide allele at each PCR amplicon was 0.034% (167amplicon) and 0.025% (198/200amplicon), suggesting minimal allelic variation. No samples contained the F167Y SNP, while one contained low-frequency reads associated with E198A (3.15%) and F200Y (3.13%). This MAD-seq method provides a highly sensitive tool to monitor the three putative benzimidazole resistance markers at individual and community levels. Further work is required to understand the association of these polymorphisms to treatment response.

## Introduction

Hookworm infections caused by *Ancylostoma duodenale, A. ceylanicum* and *Necator americanus* have a considerable impact on human health^1^, causing as many as 500 million global infections, including 198 million in sub-Saharan Africa^2,3^. Along with the other soil-transmitted helminth (STH) infections, hookworm is associated with malnutrition and anemia, as well as physical and cognitive impairment in children. Chronic disability, particularly among infected children and pregnant women, is associated with global economic losses of up to $100 billion per year^4^. Control efforts for hookworm and other STH infections rely primarily on mass drug administration (MDA) of benzimidazole anthelminthics, most commonly albendazole and mebendazole. The excellent safety record and low cost of benzimidazoles, particularly when administered through school-based programs, have resulted in the promotion of MDA as an extremely cost-effective global health intervention^5–7^. Dating back to the 2001 World Health Assembly resolution recommending targeted school-based deworming for all at-risk children, MDA has emerged as one of the largest coordinated global health initiatives ever undertaken^8,9^. Although in recent years there has been a marked decline in STH prevalence in the Americas and Asia, progress toward control and ultimate elimination of infection has been less pronounced in sub-Saharan Africa^10^.

Ghana’s most recent national survey confirms that hookworm remains endemic throughout the country, with community prevalence rates ranging between 10 and 60%^11–13^. Previous studies conducted in the Kintampo North Municipality (KNM) have demonstrated variable effectiveness of single dose albendazole against hookworm, as measured by Cure Rate (CR) and Egg Reduction Rate (ERR) following treatment^14–16^. Moreover, using an in vitro egg hatch assay, we have previously shown that exposure to benzimidazole anthelminthics may select for hookworm isolates with reduced drug susceptibility^15^. Although in vitro susceptibility was not shown to correlate with treatment response in this study, the findings raised the possibility that, like veterinary nematodes, continued exposure to benzimidazole anthelminthics could potentially lead to the emergence of genetically mediated resistance in human hookworms.

In veterinary nematodes, mutations in the isotype-1 β-tubulin gene that confer resistance to the benzimidazole anthelminthics result in structural changes associated with reduced drug binding to the target protein^17^. Previous studies aimed at detecting the presence of putative resistance markers in human STH pathogens have used molecular methods to screen genomic DNA extracted from STH eggs or larvae from infected individuals^18–21^. Among the most commonly used approaches to screen for mutations associated with anthelminthic resistance in human STH include, quantitative real-time PCR (qPCR) and pyrosequencing, both of which have limitations. Although qPCR is sensitive, high cycle threshold (Ct) values limit specificity when the quality of template gDNA is poor or the proportion of SNPs is low. In contrast, while pyrosequencing is a highly specific genotyping tool, it may lack the sensitivity to detect mutant alleles present in fewer than 5–10% of the parasite population^22,23^. More recently, amplification-refractory mutation system (ARMS)-PCR and PCR- Restriction Fragment Length Polymorphism (RFLP) has been proposed as a method more suited to screening pooled or individual egg samples for genetically mediated anthelminthic resistance^18,24–27^. These methodologies are not well suited for high throughput screening of large numbers of samples necessary for comprehensive monitoring of human hookworm populations for emerging resistance. Because of the recognized potential for repeated deworming drug exposure to elicit resistance, similar to what has been described in veterinary nematodes^28–30^, new methods are needed for effective monitoring of MDA programs in STH endemic populations. Interestingly, recent studies in veterinary nematodes have shown the potential of next-generation sequencing approaches in assessing anthelmintic resistance, which have not been yet employed in screening human STH^31,32^.

We have developed a multiplex amplicon deep sequencing (MAD-seq) approach to screen human fecal samples for the presence of hookworm (*Necator americanus*) DNA, encapsulating regions containing putative markers of benzimidazole resistance. This method provides a highly sensitive technique to measure the frequency of specific alleles within a heterogeneous population of hookworms. We describe here the application of this method using hookworm eggs collected from infected individuals in Ghana and demonstrate the capacity to identify low-frequency allelic variants within the *N. americanus* isotype-1 β-tubulin gene. By using pooled fecal samples from individuals exposed to repeated MDA, this approach is feasible for large scale screening of the three putative markers in endemic communities. More importantly, we propose the Unique Molecular Identifier (UMI)-based MAD-seq approach as a feasible, accurate, sensitive and scalable method that can be applied to other gastrointestinal parasites of both human and veterinary importance, allowing for effective monitoring of the frequency of mutations associated with anthelminthic resistance.

## Results

### Detection of *N. americanus* β-tubulin SNPs using MAD sequencing

**Figure 1:**
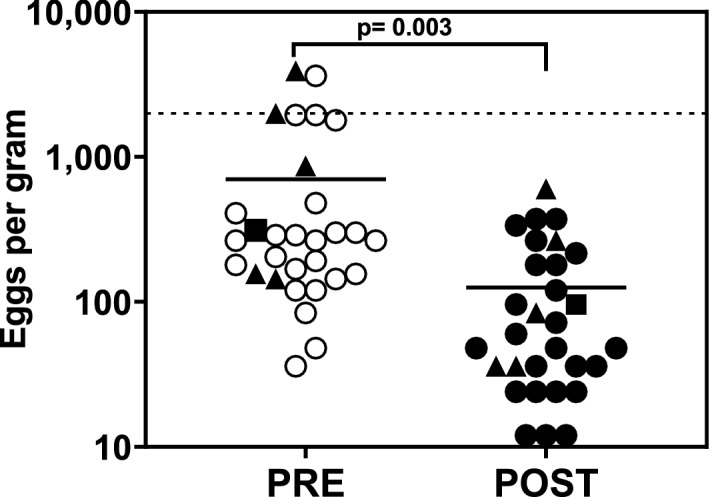
Kato-Katz microscopy was used to determine egg counts in fecal samples from each study subject before and after treatment. Each symbol represents an individual study subject. The dotted horizontal line shows the EPG threshold between light and moderate infection, as defined by WHO. The horizontal line represents the median EPG for each group. The triangles represent samples containing 2 or more amino acid residue changes present across both amplicons. The squares represent the pre- and post-treatment samples from study subject 5803, which contain both E198A and F200Y.

Among the 30 study subjects included in this analysis, single dose albendazole treatment was associated with a reduction in fecal egg counts, from a mean of 700 ± 1026 EPG (pre-treatment) to 126 ± 143 EPG (*p* = 0.003; 2-sided t-test) (Fig. 1). For each of the *N. americanus* positive samples, the targeted regions of the isotype-1 β-tubulin gene were successfully sequenced and aligned to a reference strain (accession #EF39285.1). Of the 24 million sequence reads generated, > 98% were used for further in silico analysis and showed complete coverage of the amplified loci. Read depth for all the 30 samples, pre- and post-treatment is included in Tables S1 and S2. Two study subject samples failed to return sequence data for the E198A/F200Y amplicon and were excluded from further analysis. Using UMI-generated molecular tags, each sample generated a mean (± SD) of 40,993 (± 16,089) and 82,459 (± 24,911) unique paired end reads for the 167 (Table S1) and 198/200 amplicons, respectively (Table S2). Among pre-treatment samples, there were 38,000 (± 3233) reads for the 167 amplicon and 91,600 (± 3170) reads for 198/200 amplicon, whereas for post-treatment samples, there were 43,500 (± 2955) reads for the 167 amplicon and 79,700 (± 4033) for 198/200 amplicon (Tables S1 and S2).

**Figure 2:**
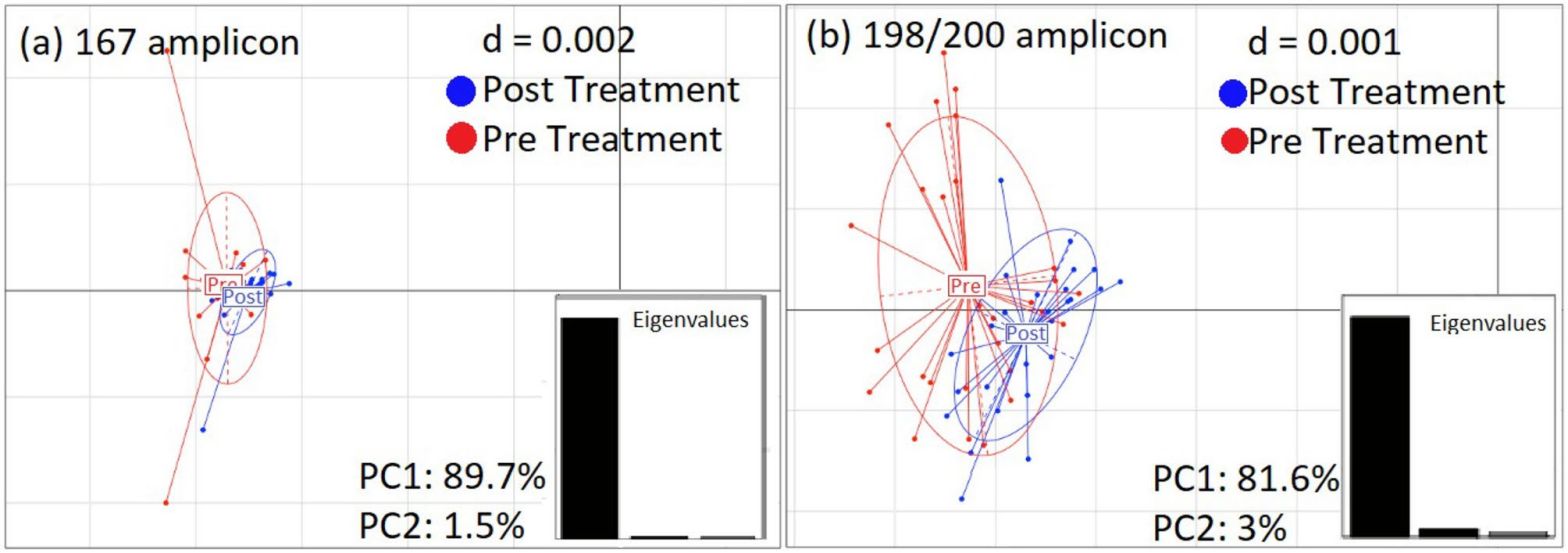
Principal component analysis of *N. americanus* isotype-1 β-tubulin DNA sequences obtained pre- and post-treatment. Eigenvalues are shown in lower right-hand corner. The first bar represents PC1, second bar represents PC2, and third bar represents PC3. Most of the values (89.7% and 81.6% for 167aa and 198/200aa, respectively) are aligned in PC1, confirming that most of the data points lie on the same axis plane, representing minimal genomic variation. Alternatively, if there were a treatment related enrichment of a particular genotype, the pre- (red) and post- (blue) treatment would not overlap, and Eigenvalues would be more evenly distributed between PC1, PC2, PC3, etc. The letter “d” represents appropriate effect size for comparison between pre- and post-treatment mean values. *d* = *((mean alternate allele frequency of post-treatment group)-(mean alternate allele frequency of pre-treatment group))/Standard Deviation.*

Principal component analysis^33^ of the relative frequencies of highest alternate alleles at each nucleotide position revealed high sequence overlap in pre-and post-treatment samples, suggesting minimal genomic variation (Fig. 2). Most of the frequency variation was captured by the first principal component (PC) (89.7%—F167Y amplicon; 81.6%—E198A/F200Y amplicon) followed by second PC (1.5%—F167Y amplicon; 3%—E198A/F200Y amplicon), as indicated by the eigenvalue graph. The highest mean alternative nucleotide allele frequency calculated for each position within the 167 amplicon was 0.034 (Table S1), while for the 198/200 amplicon it was 0.025, consistent with minimal genomic variation (Table S2).

As shown in Table 1, SNPs representing amino acid substitutions in the coding region of the β-tubulin gene were identified in amplicons from both pre- and post-treatment samples. Three samples (#5004, #7006, #5506) had mutations that resulted in variants (defined by presence of > 0.01% nucleotide substitution compared to the nucleotide in the reference strain (accession #EF39285.1)) within 3 residues of amino acid 167, which could potentially impact the drug binding pocket of β tubulin^34^. These same three samples contained variant alleles in the 198/200 amplicon reads. The presence of these mutations was not associated with response to deworming treatment among the 30 study subjects, nor were they found to be increased in post-treatment samples.

**Table 1:** Allelic variants corresponding to amino acid changes within the isotype-1 β-tubulin gene of *Necator americanus.*

Sample #	Amino acid variant			
	Amplicon 167 (N = 7)		Amplicon 198/200 (N = 13)	
	Mutation	Frequency	Mutation	Frequency
0108			(D197Y)	1.28%
0305	(S138Y)	1.37%		
0504			(Y208H)	0.54%
0804	(V170I)	0.56%		
1204	(R156Q)	0.95%		
5004	(L150F)	0.69%	(A185S)	0.63%
5506	(S168P)	20.23%	(T196I) (L207W)	0.53% 25.9%
5803			(E198A) (F200Y)	3.15% 3.13%
5909			(Y183C)	0.99%
6801	(G148V)	67.17%		
7006	(S165P)	1.13%	(S188P) (Y208C)	0.79% 0.25%

In addition to the three putative drug resistance markers at amino acid codons 167, 198 and 200, we also analyzed the sequence data for additional SNPs within the translated sequence corresponding to the β tubulin amplicons (amino acid codons 133–199; 176–229) (Table 1). SNPs within the exonic region of the F167Y amplicon included a total of 19 point mutations, of which there were 2 nonsense mutations, 7 missense mutations and 10 silent mutations. The coding sequence within the E198A/F200Y amplicon contained a total of 12 point mutations, of which 9 were missense and 3 were silent. In total there were 21 amino acid variants identified across the 2 amplicons studied using this approach.

**Figure 3:**
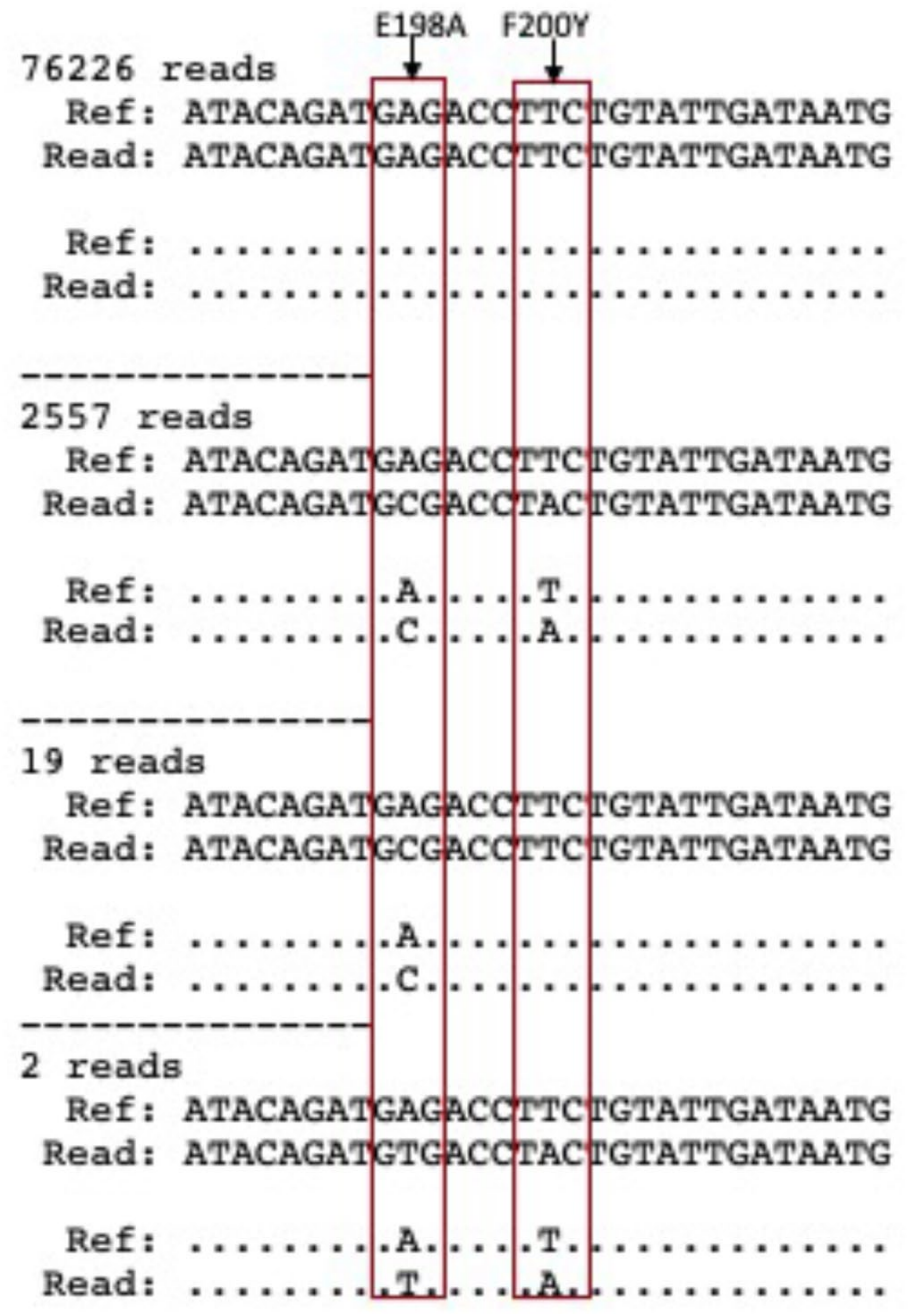
For study sample #5803, 3.2% of the total amplicon reads contained both E198A and F200Y resistance associated SNPs of the *Necator americanus* isotype-1 β -tubulin gene. Of these, 99.3% of the E198A reads also contained F200Y.

No samples contained detectable levels of the F167Y resistance allele, but one sample (Sample ID 5803; Table S2) contained reads associated with the previously reported pair of resistance associated SNPs E198A (3.15%) and F200Y (3.13%)^35^. Further sequence analysis of this sample showed that 99.3% of the sequence reads containing the aa198 GAG → G**C**G SNP also contained the TTC → T**A**C mutation at aa200 (Fig. 3), similar to what has previously been observed in *N. americanus* egg DNA samples collected from Kintampo North Municipality in the Bono East Region of Ghana^35^.

## Discussion

Despite expanded distribution of deworming drugs across sub-Saharan Africa, questions remain about the long-term impact of MDA and its effectiveness at reducing the worldwide burden of hookworm and other STHs^36–38^. Among concerns raised about the sustainability of MDA as a viable control measure is the potential for repeated exposure to variably effective benzimidazole drugs to accelerate emergence of genetically mediated drug resistance in communities where STH infections remain prevalent^39–42^. Much of the concern has been driven by the example of veterinary nematodes, which routinely develop resistance to various classes of anthelminthic following repeated exposure^23,43–45^. Although evidence of widespread drug resistance in human STH is lacking, at present there is no universally accepted standard for assessing or monitoring for resistance in endemic populations. In addition, countries that utilize MDA for STH control typically lack access to the technology and resources needed to implement programs that can detect drug resistance as it emerges.

Data from the Kintampo North Municipality (KNM) in the Bono East Region of Ghana, where government-sponsored MDA has been in place since 2007, highlight the challenges of hookworm control in sub-Saharan Africa. Numerous cross-sectional epidemiological studies in this region have documented variable effectiveness of albendazole treatment, based on standard metrics of egg reduction rate (ERR) and cure rate (CR)^15,16,46^.

The veterinary nematode experience suggests that the expansion of MDA across human populations could lead to emerging resistance, thereby affirming the need to monitor for known and potentially novel mutations in drug targets from hookworm and other STHs. We have developed a highly sensitive targeted approach to analyze genetic variation of three putative markers within the hookworm (*Necator americanus*) isotype-1 β-tubulin gene, the molecular target of benzimidazole anthelminthics. This MAD-seq method was used to analyze pre- and post-treatment hookworm egg samples from 30 study subjects using a UMI-based approach. The advantage of this method compared to other techniques is the capacity to analyze pooled eggs collected from multiple individuals in a limited number of sequencing reactions, making it amenable to screening large numbers of samples at once. The approach is also capable of detecting very low levels of allelic variants, as shown here. More importantly, we demonstrate that the MAD-seq method can simultaneously perform in-depth genomic analysis of multiple targets. This method, therefore, could also be modified to assess putative resistance markers identified in the future by simply designing new PCR primers.

Principle Component Analysis of sequence data from the Kpandai samples revealed minimal difference between the pre- and post-treatment samples, suggesting that the single dose of albendazole did not enrich for a specific hookworm genotype, based on the limited sequence of the isotype-1 β-tubulin gene that was amplified. However, the method was sensitive enough to detect low level heterogeneity across the two amplicons, with mean alternative allele frequencies of 0.034% (PCR amplicon containing aa167) and 0.025% (PCR amplicon containing aa198/200). We also found low mean alternative allele frequencies for the 3 putative markers of drug resistance: F167Y (0.031%), E198A (0.021%) and F200Y (0.023%). To prevent the introduction of any amplification bias introduced by PCR and sequencing, similar to the work reported by Avramenko et al.^31^ we used a conservative approach. This involved using an in silico method to identify sequencing reads that vary > 0.01% at each nucleotide position in comparison to the reference genome and then focusing on those reads that exhibited > 0.5% nucleotide variation. The ability of MAD-seq to delineate subtle differences across relatively short reads demonstrates the potential of this method as a tool to monitor changes in hookworm genotypes over time and across different geographic regions, as well as assess changes in hookworm populations resulting from repeated deworming drug exposure.

Further analysis of sequence data identified low-frequency SNPs in the DNA sequences corresponding to amino acid residues 133–199 and 176–229 of the *N. americanus* β-tubulin protein. Across the two amplicons, 35 SNP mutations corresponding to 20 amino acid substitutions were detected in a total of 12 study subject samples. The amino acid substitutions identified in amplicon 1 (aa167) occurred in seven individual samples, while 4 samples contained multiple SNPs in amplicon 2 (aa198/200). Three study subject samples contained amino acid substitution mutations in both amplicons. Although no samples contained detectable levels of the F167Y resistance allele, one sample contained reads with the E198A (3.15%) and F200Y (3.13%) mutations in tandem. The combination of these two resistance associated SNPs was previously identified in hookworm egg samples from Kintampo North, Ghana^35^. The degree to which co-segregation of these two SNPs impacts benzimidazole effectiveness is unclear, although point mutations in 2 of the 3 amino acid residues critical for the binding of drug to hookworm isotype-1 β-tubulin could potentially alter protein structure and reduce albendazole susceptibility^17^. Importantly, detection of these SNPs in hookworm parasites collected from geographically distinct regions of Ghana (Kintampo North and Kpandai) warrants further investigation to understand the association of these polymorphism to treatment response and highlights the need for monitoring endemic communities for potential resistance-associated genotypes.

Despite the fact that school age children living in the study area have been subjected to more than a decade of repeated rounds of government sponsored MDA with albendazole^47^, the overall response to drug treatment was favorable (Fig. 1), which is consistent with the low frequency of resistance-associated SNPs identified in this study. With an ERR of 89% across the study population, the data from Kpandai differ from prior studies conducted in Kintampo North Municipality, where albendazole appears to be less effective against hookworm^14–16^. Interestingly, prior studies in Kintampo North using the SNP-specific qPCR method demonstrated the presence of F167Y and E198A/F200Y resistance-associated mutations at much higher frequencies than reported here in Kpandai^35^. Taken together, the data from these two studies suggest that within Ghana there is heterogeneity in both the effectiveness of deworming drug treatment and the effect of drug exposure on the prevalence of putative resistance markers among *N. americanus* hookworms.

Over the past decade, the reach of MDA programs for STH control has expanded due to renewed commitments from global pharmaceutical companies, NGOs, foundations, and government Ministries of Health. In addition, there have been recommendations to expand the number of groups eligible for targeted deworming, such as pre-school age children and pregnant women^48^. More recently, it has been suggested that elimination of hookworm may not be possible without community-wide MDA, which by including adult populations would significantly increase the number of individuals exposed to benzimidazoles within endemic communities, as well as globally^48^. The sustainability of MDA as a control or elimination strategy must address the potential for emerging drug resistance, which has been well documented in veterinary helminths but to date has not been identified as a cause of reduced deworming drug effectiveness in humans.

Ideally, effective monitoring of communities subjected to MDA with benzimidazole anthelminthics that are at risk for emerging resistance would include the capacity to detect low frequency alleles associated with reduced drug susceptibility prior to the loss of clinical effectiveness. To be cost-effective in resource-limited settings, screening methods must be sensitive enough to identify variants in pooled samples that are representative of a household, village or larger community, aiding to improve critical surveillance capabilities needed to guide policy decisions. Future work will be aimed at defining the accuracy and cost-effectiveness of this method as a tool to monitor for emerging benzimidazole resistance in Ghana and other hookworm endemic communities.

At present there are no routinely available field-adapted methods for detecting putative benzimidazole resistance markers that are both sensitive and scalable. The MAD-seq protocol reported here could feasibly, and with modest additional cost, be adapted to analyze hundreds of pooled samples in a relatively small number of reactions, thereby facilitating the screening of putative resistance markers at the community level. Importantly, this method could also be adapted to screen for allelic variants in other STHs, including *Ascaris lumbricoides* and *Trichuris trichiura*, providing access to valuable information that can be used to inform neglected tropical disease control program policies and practice.

## Methods

### Sample selection and hookworm egg DNA preparation

In a cross-sectional field study of hookworm infection carried out in Kpandai District, Ghana, between June and August 2016, a total of 111 individuals were found to be hookworm positive at baseline using Kato-Katz microscopy, for an initial prevalence of 19.6%. Among the 97 who were tested following single dose oral albendazole (400 mg) treatment, 34 (35%) remained infected. Matched pre- and post-treatment hookworm egg samples were available for subsequent analysis from 30/34 subjects (88%) who were not cured. Pretreatment egg counts in 28 of the 30 study subjects (93%) were classified as “light” intensity (1–1999 hookworm eggs per gram (EPG) of feces) according to WHO guidelines^49^, while two samples contained moderate intensity infection (2000–3999 EPG). All post-treatment samples were classified as light infections. Prior to enrolling subjects, ethical clearance for the field study was approved by the Yale University Human Investigation Committee (Protocol #1,304,011,926) and Noguchi Memorial Institute for Medical Research (NMIMR) Institutional Review Board (Study #115/15–16). The hookworm eggs isolated from faecal samples were de-identified prior to being shipped to Yale School of Medicine, USA from Noguchi Memorial Institute of Medical Research, Ghana where consent was obtained from study participants.

Genomic DNA was extracted from hookworm eggs collected from those 30 subjects who continued to show persistent infection following treatment. Hookworm eggs were purified from each fecal sample on the day of collection by sequential sedimentation, flotation and filtration as previously described^15,50^. Egg samples were stored in RNAlater (Invitrogen) solution and kept frozen until further analysis. Genomic DNA was isolated from purified egg samples using the QIAamp DNA stool mini kit (QIAGEN) according to the manufacturer’s protocol and stored at − 20 °C.

### Primer design and amplicon sequencing

**Figure 4:**
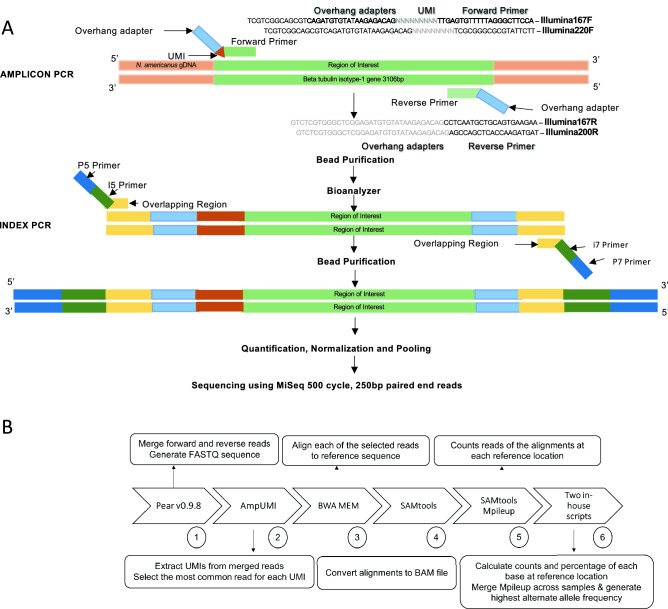
(**A**) Workflow illustrating UMI based methods for amplifying individual strands of genomic DNA within pooled samples of hookworm eggs. (**B**) Workflow illustrating in silico analysis of next generation sequencing data representing pre- and post-treatment hookworm egg samples.

Extracted Genomic DNA from individual fecal samples was used as template in 2 separate PCR amplifications capturing the three putative resistance markers by targeting the surrounding genomic region as shown in Fig. 4A. The 167 amplicon (226 bp) corresponds to the F167Y mutation in exon 4, while the 198/200 amplicon (262 bp) captures the E198A and F200Y SNPs in exon 5 of the β-tubulin isotype-1 gene of *N. americanus*^21,51–53^. Two sets of forward and reverse primers were designed using Geneious Prime (Dotmatics), followed by sanger-sequencing to confirm absence of any non-specific amplification. A Unique Molecular Identifier (UMI) of 9 degenerate oligonucleotides was then added to the 5’ end of each forward primer. (F167Y forward—5’ TCGTCGGCAGCGTCAGATGTGTATAAGAGACAGNNNNNNNNNTTGAGTGTTTTTAGGGCTTCCA 3’; F167Y reverse – 5’ GTCTCGTGGGCTCGGAGATGTGTATAAGAGACAGCCTCAATGCTGCAGTGAAGAA 3’; E198A/F200Y forward – 5’ TCGTCGGCAGCGTCAGATGTGTATAAGAGACAGNNNNNNNNNTCGCGGGCGCGTATTCTT 3’; E198A/F200Y reverse – 5’ GTCTCGTGGGCTCGGAGATGTGTATAAGAGACAGAGCCAGCTCACCAAGATGAT 3’). The UMI was then incorporated into each individual strand of gDNA, facilitating subsequent molecular analysis. The primer design also incorporated standard dual-index sequencing adapters for MiSeq workflows into both forward and reverse primers.

Hookworm genomic DNA was combined with forward and reverse primers and KAPA HiFi HotStart Ready Mix (Roche Molecular Systems). Thermocycling conditions (95 °C for 5 m; 12 cycles of 95 °C for 30 s, 65 °C for 30 s, 72 °C for 30 s; 33 cycles of 95 °C for 30 s, 56 °C for 30 s, 72 °C for 30 s; 1 cycle of 60 °C for 30 m) were optimized prior to purification of DNA using paramagnetic beads. The PCR products were quantified, and size verified (167amplicon: 226 bp; 198/200amplicon: 262 bp). Nextera Index Barcodes were then added to both amplicons of a matched set for the 30 pre- and post-treatment gDNA samples in a brief PCR reaction (95 °C for 3 m; 8 cycles of 95 °C for 30 s, 55 °C for 30 s, 72 °C for 30 s; 72 °C for 5 m). A step-by-step schematic for amplifying, barcoding and pooling is shown in Fig. 4A.

### Pooling and sequencing of libraries

The DNA concentration of each final Index PCR product was quantified using a fluorometric method (Qubit 3.0, ThermoFisher), and samples were diluted to a final concentration of 10 nM each before pooling. The pooled library was sequenced using a MiSeq Illumina desktop sequencer and 500-cycle paired-end reagent kit (MiSeq Reagent Kit v2; Catalog #MS-102-2003; final concentration of 15 pM). The PhiX control v3 (15%; Illumina, FC-110-3001) was added due to low diversity created by targeted amplicon sequencing. Collected sequencing data were uploaded and made available from EMBL Nucleotide Sequence Database repository (SRA: PRJNA753088).

### Sequence analysis

An in silico process was used to analyze the sequencing data obtained from matched pre- and post-treatment egg DNA samples (Fig. 4B). PEAR v. 0.9.8 software was first used to merge the forward and reverse reads for each paired-end read in order to generate contiguous FASTQ sequences for subsequent analysis^54^. Following this initial step, AmpUMI^55^ was used to extract the UMI's from each merged read, to group the reads by UMI sequence, and to select the most common read found for each UMI. BWA-MEM software was then used to align each of the selected reads to the reference (accession #EF39285.1) sequence^56^, and SAMtools was used to convert the alignments into a BAM file^57^. SAMtools Mpileup^58^ was then used to count the read alignments at each nucleotide location of the reference gene, and an in-house script was designed to define the counts and highest alternate nucleotide percentage of each base at the respective reference location. In addition, a custom script was designed to merge the Mpileup results across all samples, generating the final alternate allele percentage for each sample at every reference location.

### Statistical analysis

Differences between groups were analyzed using a 2 tailed t-test. Principal component analysis (PCA) was performed using R studio (Version 1.2.5019; Package ‘Ade4’). We transformed the highest alternate allele percentages at each nucleotide, which represents a set of correlated variables, into a substantially smaller set of uncorrelated variables. This PCA-based approach allowed us to consider all the data from both pre- and post-treatment samples from 167 amplicon and 198/200 amplicon.

## Supplementary Information

Supplementary Information 1.

Supplementary Information 2.

## Data Availability

The sequencing raw reads generated and analyzed in the current study are available in EMBL Nucleotide Sequence Database repository (SRA: PRJNA753088).
